# *Mycoplasma pneumoniae* as a cause of vulvar ulcers in a non-sexually active girl: a case report

**DOI:** 10.1186/s13256-017-1345-9

**Published:** 2017-07-09

**Authors:** Maria G. Koliou, Talia Kakourou, Jan Richter, Christina Christodoulou, Elpidoforos S. Soteriades

**Affiliations:** 1 Department of Paediatrics, Archbishop Makarios III Hospital, Strovolos, Nicosia Cyprus; 2 University of Cyprus, School of Medicine, Nicosia, Cyprus; 3 Cyprus Institute of Biomedical Sciences, Nicosia, Cyprus; 4 First Pediatric Department, University of Athens School of Medicine, Athens, Greece; 5 Department of Molecular Virology, Cyprus Institute of Neurology and Genetics, Nicosia, Cyprus; 6 Harvard School of Public Health, Department of Environmental Health, Environmental and Occupational Medicine and Epidemiology (EOME), Boston, USA

**Keywords:** *Mycoplasma pneumoniae*, Acute vulvar ulcers, Non-sexually active females, Cyprus

## Abstract

**Background:**

Non-sexually active young females very rarely develop genital ulcers. Such ulcers pose a diagnostic challenge as well as physical and emotional distress for patients and family; therefore, the search for their etiology requires exhaustive investigation. Several viruses such as Epstein–Barr virus have been associated with this entity; however, *Mycoplasma pneumoniae* has rarely been linked to such ulcers in the literature. We present a case of vulvar ulcers in a non-sexually active young girl during the course of pneumonia caused by *Mycoplasma pneumoniae*.

**Case presentation:**

A 10-year-old non-sexually active girl of cypriot origin presented at a hospital with fever, dry cough, and acute vulvar ulcers. Laboratory investigations as well as imaging studies revealed *Mycoplasma pneumoniae* as the cause of her pneumonia and acute vulvar ulcers.

**Conclusions:**

Although a rare cause of vulvar ulcers, *Mycoplasma pneumoniae* should be considered in the differential diagnosis of acute vulvar ulcers coexisting with respiratory symptoms.

## Background

Non-sexually active young females very rarely develop genital ulcers. However, such ulcers pose a significant diagnostic challenge and they may cause physical and emotional distress for patients and families since they may raise suspicion of possible sexual abuse and/or venereal etiology. Therefore the search for the etiology of genital ulcers requires exhaustive investigation. Despite the above challenges, a definite diagnosis of the cause of such ulcers often cannot be identified.

In 1913, Lipschütz described vulvar ulcers in adolescent and virgin females where no specific cause was detected [1]. Since this first description, several additional possible causes have been reported in the literature. In most cases, symptoms consistent with a viral illness precede the development of the ulcers and the differential diagnosis includes infectious agents [2]. Several case reports and case series have associated these vulvar ulcers in non-sexually active females with viral infections such as Epstein–Barr virus [3], cytomegalovirus (CMV), influenza A virus, mumps virus [4, 5], and in some cases with autoimmune disorders such as Behçet’s disease [6] or trauma [7]. Herpes simplex virus (HSV) has been associated with such ulcers, most commonly in sexually active patients. *Mycoplasma pneumoniae* has rarely been linked with these ulcers in the medical literature [8, 9].

*Mycoplasma* species represent the smallest bacterial cells discovered. Several species are pathogenic in humans, the main one being *M. pneumoniae,* which is mainly responsible for respiratory tract infections. Extrapulmonary manifestations, such as dermatologic, can also occur with or without concurrent respiratory infection in up to 25% of human *Mycoplasma* infections. These include nonspecific erythematous maculopapular rashes, Stevens–Johnson syndrome, Henoch–Schönlein purpura, and mucositis [10].

In this report we describe the case of a young non-sexually active girl who developed acute vulvar ulcers during the course of pneumonia caused by *M. pneumoniae*.

## Case presentation

A 10-year-old girl of cypriot origin was referred to hospital because of fever and ulcers in the genital area. Three days before admission she started having fever, sore throat, and mild dry cough. A quick pharyngeal test for group A streptococcal antigen was found negative. On day 3 after the onset of fever, she developed intense pain and burning in her genital area for which she was admitted to our hospital.

**Fig. 1:**
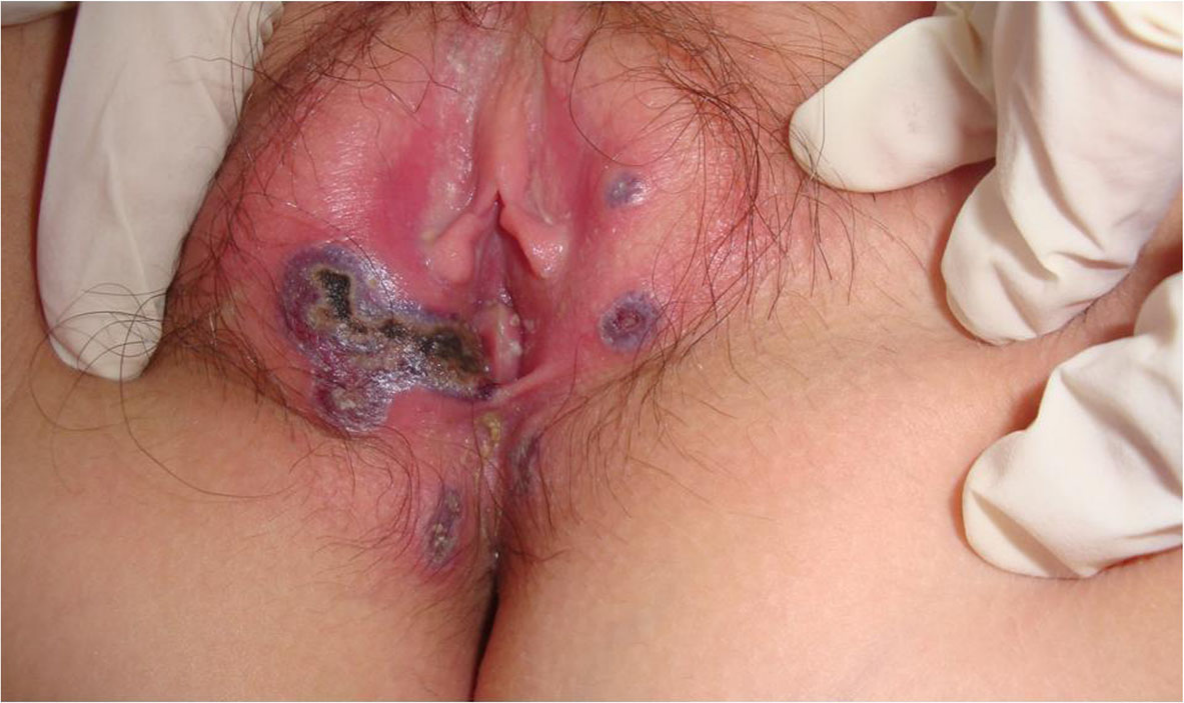
Genital ulcers of the patient

On admission, she had fever and cough but no diarrhea or abdominal pain. On examination, painful ulcers of variable depth and diameter with elevated violaceous borders located inside the labia majora were noted (Fig. 1). No oral ulcers or swollen lymph nodes were detected. She was not sexually active and had no previous history of similar ulcers. She was initially treated with intravenously administered antibiotics and local wound care with antiseptics.

**Fig. 2:**
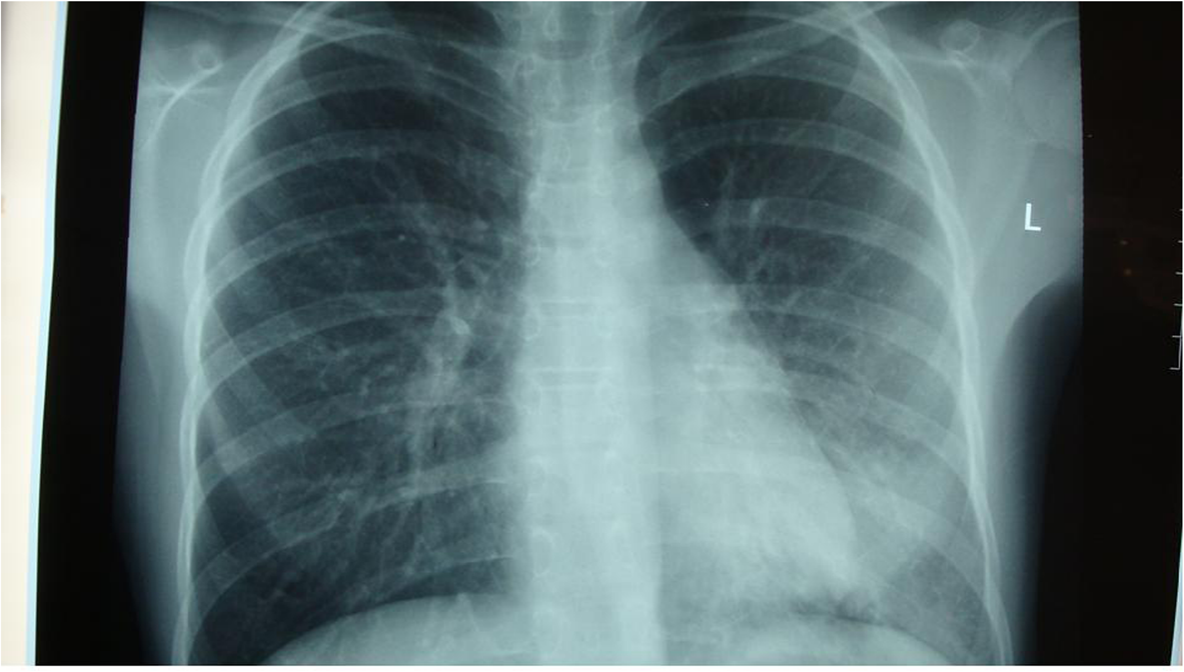
Chest X-ray revealing right lower lobe pneumonia

Cultures from the lesions were negative for bacteria. Polymerase chain reaction (PCR) in the tissue for HSV was also negative. Antibodies against HSV1 and HSV2, antinuclear antibodies, anti-double-stranded deoxyribonucleic acid (dsDNA), antineutrophil cytoplasmic antibodies (ANCA)-proteinase (PR3), and ANCA-myeloperoxidase (MPO) were all negative. Four days after admission the lesions significantly improved with local treatment, but her fever and cough persisted. A chest X-ray revealed a right-sided pneumonia (Fig. 2). Orally administered azithromycin was added as a treatment agent and 2 days later she became afebrile. *Mycoplasma* immunoglobulin M (IgM), as measured by a semi-quantitative enzyme-linked immunosorbent assay (ELISA) method on day 11 after onset of symptoms, was found to be highly positive. Three weeks later, *Mycoplasma* IgM titer was also found positive but at a lower titer. Real-time PCR for *M. pneumoniae* in nasopharyngeal secretions was positive and strongly positive in two consecutive sputum specimens. However, PCR for *Mycoplasma* in the tissue specimen from the genital lesions was found negative.

For the detection of the bacteria, a duplex real-time PCR assay was employed targeting the Community Acquired Respiratory Distress Syndrome (CARDS) toxin gene of *M. pneumoniae* and the major outer membrane protein gene of *Chlamydia pneumoniae*. In addition, the respiratory samples were tested for a panel of respiratory viruses without yielding a positive result: influenza A and B, respiratory syncytial virus (RSV), rhinovirus, parainfluenza viruses 1 to 4, adenovirus, human metapneumovirus, human bocavirus, enterovirus, and human coronaviruses 229E, OC43, and NL63. During a 2-year follow-up after hospital discharge, she had no recurrence of the ulcers.

## Discussion

*M. pneumoniae* has been linked to acute vulvar ulcers in a few previous reports in the literature. In most of these reports the diagnosis was established by measuring IgM-specific antibodies against the *Mycoplasma*. In our case, both serology and molecular methods were used in order to diagnose acute *M. pneumoniae* infection.

A culture of *M. pneumoniae,* despite being the gold standard method for diagnosis, usually has a mean incubation period of 10 to 14 days and low sensitivity. Therefore, clinical diagnosis relies mainly on serology and in recent years also on molecular methods. Detection of anti-*Mycoplasma* IgM antibody usually indicates acute infection. In adults, IgM antibodies may not develop and only an immunoglobulin G (IgG) response can be detected, especially in cases of re-infection [11]. However, this is not the case in children where past infection by *M. pneumoniae* is unlikely and therefore measurement of IgM is considered a quite reliable method for diagnosis.

The direct detection of *M. pneumoniae* nucleotide sequences from clinical specimens such as sputum, nasopharyngeal aspirate, or pharyngeal swab, has been proven to be a very sensitive and specific approach, which offers a rapid diagnosis of infection [12, 13]. However, in some cases, detection of *M. pneumoniae* from respiratory tract secretions may only indicate carriage [10]. The utilization of a combined approach of serology with PCR techniques, if available, may surpass the limitations of either technique when used alone.

The diagnosis of *M. pneumoniae* infection in our case was based on both positive IgM antibodies and the detection of the genetic material of the pathogen by real-time PCR. A possible causative mechanism for these extrapulmonary manifestations is the immune-mediated damage against host tissue caused by the variety of cross-reactive antibodies, which are generated as a result of *M. pneumoniae* infection [10]. However, in some cases *M. pneumoniae* has been isolated directly from the lesions [14]. In these cases, direct damage of the tissue by the pathogen is implicated. In our case, neither *Mycoplasma* nor any other pathogen was isolated directly from the skin lesions, therefore the immune-mediated effect may be the most probable explanation.

When these ulcers develop in an adolescent, the investigation should be based on a careful and non-offensive sexual history. The differential diagnosis should include infections that may or may not be sexually transmitted such as HSV, syphilis, chancroid, and human immunodeficiency virus (HIV) as well as Epstein–Barr virus, CMV, influenza A virus, and *M. pneumoniae.* In cases of recurrent genital manifestations combined with or without oral ulcers, the possibility of systematic autoimmune diseases such as Behçet’s or aphthosis should also be considered [15].

In most cases of vulvar ulcers in non-sexually active females, the disease is usually self-limited and local care is sufficient. Systemic use of anti-bacterial drugs or anti-viral drugs does not appear to alter the course of the disease [2]. This was also obvious in our case where her skin lesions significantly improved with local care while the *M. pneumoniae* infection was still active and required the administration of antimicrobial treatment such as azithromycin.

## Conclusions

*M. pneumoniae* is a rare cause of acute genital ulcers in non-sexually active young females. It should be considered in the differential diagnosis especially if the ulcers are accompanied or preceded by symptoms from the respiratory tract. Despite the fact that vulvar ulcers in non-sexually active young females are a benign and self-limited condition, investigation of the cause of illness should exclude potential sexually transmitted diseases and could reassure both the patient and the family.

## Abbreviations

ANCA: Antineutrophil cytoplasmic antibodies
CARDS: Community Acquired Respiratory Distress Syndrome
CMV: Cytomegalovirus
dsDNA: Double-stranded deoxyribonucleic acid
ELISA: Enzyme-linked immunosorbent assay
HIV: Human immunodeficiency virus
HSV: Herpes simplex virus
IgG: Immunoglobulin G
IgM: Immunoglobulin M
MPO: Myeloperoxidase
PCR: Polymerase chain reaction
PR3: Proteinase 3
RSV: Respiratory syncytial virus
